# Mortality-related risk factors and long-term survival after 4460 liver resections in Sweden—a population-based study

**DOI:** 10.1007/s00423-016-1512-2

**Published:** 2016-10-01

**Authors:** Stefan Gilg, Ernesto Sparrelid, Bengt Isaksson, Lars Lundell, Greg Nowak, Cecilia Strömberg

**Affiliations:** 1 Institution for Clinical Science, Intervention and Technology (CLINTEC), Karolinska Institutet, Solna, Sweden; 2 Department of Surgery at Centre for Digestive Diseases, Karolinska University Hospital, Stockholm, Sweden; 3 Department of Transplantation Surgery, Karolinska University Hospital, Stockholm, Sweden

**Keywords:** Hepatectomy, Outcome, Long-term survival, Risk factors, Population based

## Abstract

**Purpose:**

The objectives of this study were to analyze the outcome after hepatectomy and to identify contributing factors to mortality and long-term survival in a population-based setting.

**Method:**

A retrospective, nationwide register study was performed. All patients who underwent hepatectomy in Sweden between 2002 and 2011 were identified in the Swedish Hospital Discharge Registry using their unique personal identification numbers. This cohort was linked to the National Cancer Registry (cancer diagnosis), the National Registry of Causes of Death, and the Migration Registry. Survival analysis by Kaplan-Meier method was performed to assess long-term outcome. A Cox regression model was used to analyze risk factors affecting long-term survival.

**Results:**

Overall, 4460 hepatectomies were performed. The 30- and 90-day mortalities were 1.8 and 3.1 %, respectively. The overall 5- and 10-year survival rates for all diagnoses were 45 and 38 %, respectively. Independent risk factors for 5-year mortality were as follows: patient age, comorbidity, male gender, intrahepatic/extrahepatic cholangiocarcinoma, gallbladder cancer, extent of hepatectomy, and hepatectomies performed at non-university hospitals. Re-resection (78.1 % with diagnosis “metastasis”) was performed on 374 patients. In these patients, mortality risk decreased by >50 % (HR 0.42; 95 %, CI 0.33–0.53).

**Conclusion:**

In a population-based analysis, liver resections are done with a low mortality risk and good long-term outcome. Patients who underwent resection at a University Hospital showed a significant better outcome compared to patients resected at non-University Hospitals. These results support further centralization of liver surgery. Re-resection should be performed if feasible.

## Introduction

During the last 20 years, liver surgery has undergone substantial changes mainly due to technical and medical innovations and is considered to offer the best opportunity of cure in cases of primary as well as secondary liver tumors [1–4]. Accordingly, the number of hepatectomies carried out has been constantly growing concomitantly with a centralization of these procedures into high volume academic centers [5, 6]. A variety of factors have been identified as contributing to decreased postoperative mortality rates and improved long-term survival rates [7–10]. However, the current literature is biased by a predominance of single-institution studies emerging from highly specialized centers, and the results presented do not necessarily reflect population-based short- and long-term results [11–13]. Population-based studies on overall mortality and long-term survival after hepatectomy are sparse. Therefore, it is uncertain to which degree the implementation and dissemination of changes in liver surgery has had any impact on the general population. Mortality related to liver resections seems to be lower when performed at high-volume, academic centers compared to low-volume, non-academic centers [14, 15], and surgeon training has been found to be highly predictive for post-operative complications [16]. This was supported by a recently published population-based study where Farges and colleagues concluded that reported mortality figures in the literature probably underestimate the real mortality risk associated with liver resection when population-based data were carefully analyzed [15, 17].

In Sweden, population-based data are available for analyses on both short-term results as well as long-term survival after all surgical procedures by utilizing the national medical registries [6, 18]. The objectives of the present study were to analyze both the incidence and which factors may have influenced the outcome of liver surgery in Sweden between 2002 and 2011.

## Patients and methods

All patients who underwent hepatectomy in Sweden during a 10-year period (2002–2011) and have been registered in the Swedish Hospital Discharge Registry were included in this cohort study. This registry was founded in 1965, and since 1987, it includes all in-hospital patient contacts; these can be traced through the patients’ national registration number for identification. The register contains both medical data (e.g., diagnosis, comorbidity, and procedure code) and general patient-related data (e.g., age, sex) but no specific information of, e.g., patient medication. The National Cancer Registry was set up in 1957 and collects cancer-related data like tumor site, histological type of cancer, and date of diagnosis but no specific pathological data like resection margins or lymph node status. In addition, the Registry of Causes of Death contains the individuals’ death certificates with information such as underlying disease and date of death. All registers are endorsed and maintained by the Swedish Board of Health and Welfare. For this specific study, we identified all patients by their unique national registration numbers in the Hospital Discharge Register while matching an in-hospital discharge procedure code for liver resection according to the Tenth Revision of the International Classification of Diseases and Procedures (ICD 10 codes JJB00, JJB10, JJB20, JJB30, JJB40, JJB50, JJB53, JJB60, JJB71, and JJB96). Then, the personal national registration number was used for cross-linkage with the Registry of Causes of Death to estimate postoperative as well as long-term survival outcomes, and the Registry of Domestic and International relocations was used for censoring in the event of a cohort member emigrating. The cohort was followed until December 31, 2011. A detailed description of the methods used in this study has been described elsewhere [19].

In the risk factor analysis, the variable age was categorized into quartiles: ≤54, 55–63, 64–71, and ≥72 years, with the study period divided into two 5-year intervals. The individual patient’s comorbidity was classified according to the Charlson score [20], modified due to the fact that almost all patients had a diagnosis of malignancy, which was excluded from the score. The respective variables were categorized into four groups: no comorbidity, Charlson score 1–2, Charlson score 3–4, and Charlson score ≥ 5.

All liver resections, both open and laparoscopic, were subdivided into three groups: minor (≤ 2 Couinaud segments), major (3–4 Couinaud segments), and extended (>4 Couinaud segments). Biopsies, ablations, and de-roofing of liver cysts were excluded from the analysis. All identified patients were stratified for diagnosis as stated in the discharge record as a surrogate for the indication for surgery. Accordingly, we identified the following diagnoses: metastases (ICD10 code C78.7), hepatocellular cancer (HCC, ICD10-code C22.0), intrahepatic (ICC, ICD10-code C22.1) and extrahepatic bile duct cancer (ECC, ICD10-code C24.0), gallbladder cancer (GBC, ICD10-code C23.9), and others/unclear. The patients with unclear diagnoses were then cross-linked with the National Cancer Register and subsequently classified into the corresponding identified group. A diagnosis of colorectal cancer (ICD10-codes C18.0-C18.9, C19.9, C20.9) along with a procedure code for liver resection was analyzed separately. The remaining patients were categorized as either benign or indefinite diagnosis. Hospitals were categorized into non-university and university hospitals as well as high-volume (>300 resections during the study period) and low-volume centers.

For the risk factor analysis, hospitals were categorized as high- and low-volume hospitals (cutoff 300 resections during the study period), as well as university and non-university hospitals (7 university, 33 non-university hospitals), respectively. The Regional Research Ethics Committee of Stockholm approved the study protocol (DN 2010/1872-31/2).

### Statistical analysis

Several patients had surgical liver procedures registered at more than one time point, and each procedure was handled as a separate event. Data were calculated as means ± standard deviations for continuous variables, and proportions for categorical variables. Long-term survival rates after liver resection were assessed by the Kaplan-Meier method.

Cox proportional hazard ratios (HRs) with 95 % confidence intervals (CI) were used for univariable and multivariable assessments of the association between potential risk factors and the hazard; in other words, the risk of death of all causes with time-at-risk as the underlying timescale was used. Potential risk factors used in the regression modeling were categorized in order to facilitate the analyses. Introducing the variables stepwise into the multivariable regression model tested potential confounding effects, and the risk factors were also tested for possible statistical interactions. *P* values <0.050 were considered to be statistically significant. Statistical analyses were performed using SPSS Version 20 for Windows (SPSS, Inc., Chicago, IL).

## Results

**Fig. 1:**
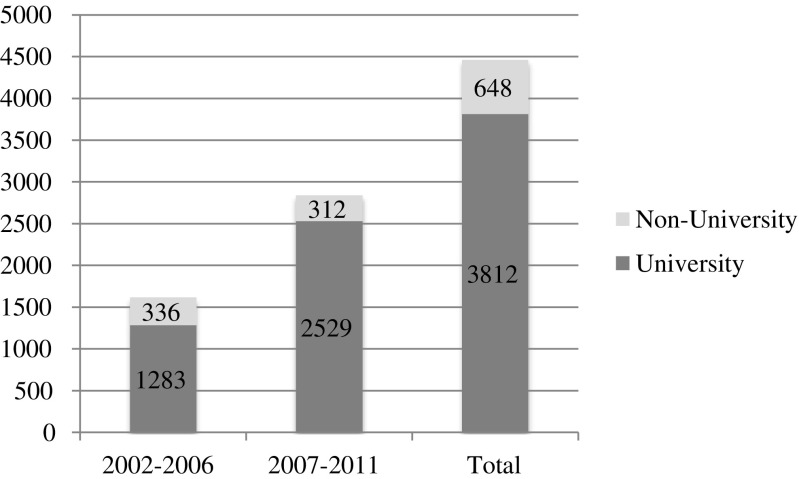
Number of liver resections in university hospitals (*n* = 7) and non-university hospitals (*n* = 33) in Sweden over the study period

**Fig. 2:**
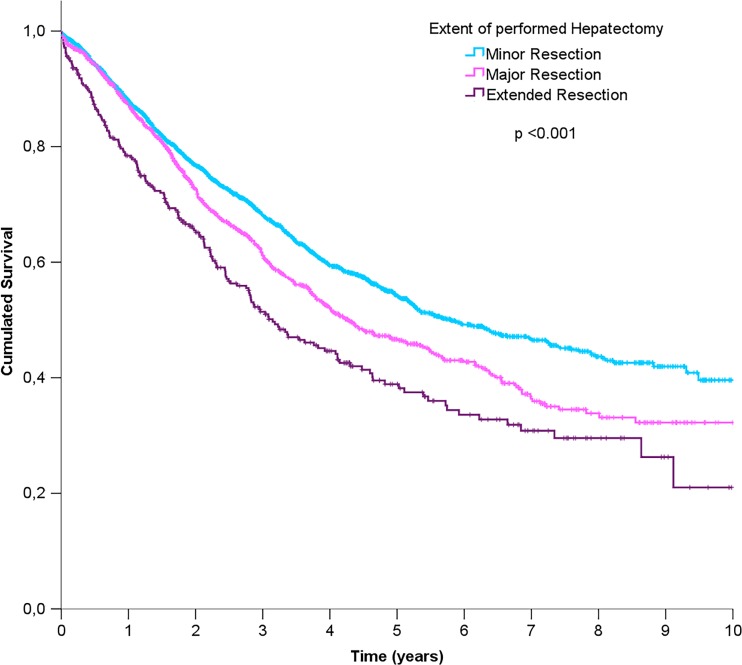
Kaplan-Meier survival estimates for minor, major, and extended hepatectomy

**Fig. 3:**
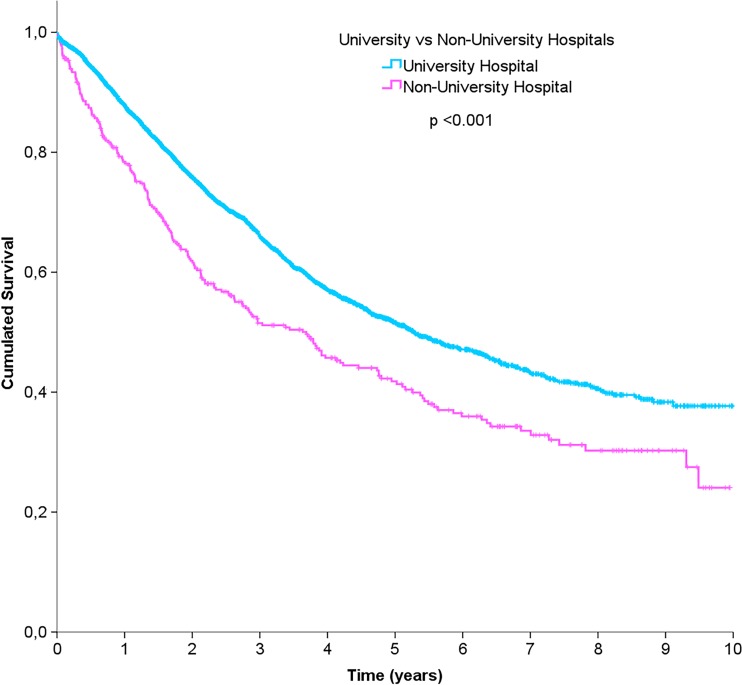
Kaplan-Meier survival estimates for hepatectomies performed at university vs non-university hospitals

**Fig. 4:**
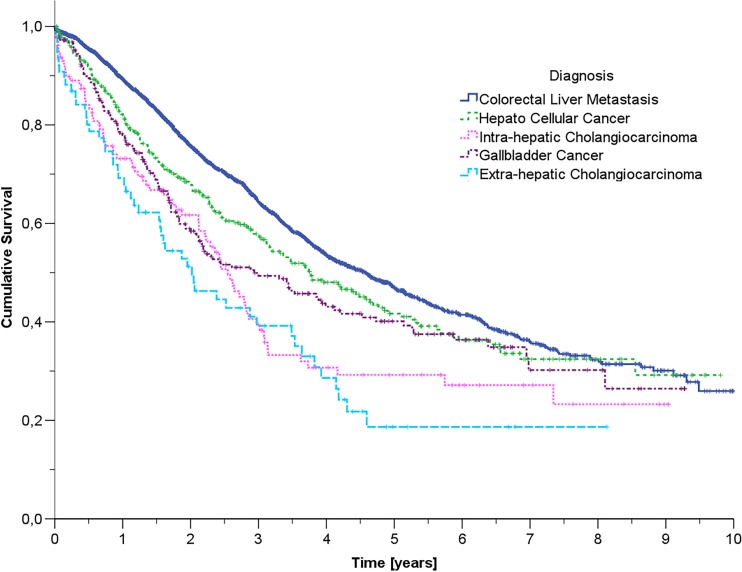
Kaplan-Meier surival estimates for different malign diagnosis

During the study period, there was an increase in annual number of liver resections. In total, 4460 (2381 (53.4 %) female, 2079 (46.6 %) male) patients with a median age of 64 were submitted to hepatic surgery. Of these, 374 patients underwent re-resection. Hepatectomies were continuously performed in 40 hospitals over the entire study period. The proportion of liver resections performed in academic compared to non-academic units is shown in Fig. 1. The incidence of hepatic resections increased from 2.5 per 100,000 inhabitants in 2002 to 8.1 per 100,000 inhabitants in 2011. At the same time, the number of minor resections increased from *n* = 1013 to *n* = 1832, major resections from *n* = 479 to *n* = 791, and extended resections from *n* = 127 to *n* = 218, when comparing the first and second 5-year period. Major/extended hepatectomies were almost exclusively performed at academic hospitals (96 % during the first 5-year period and 99 % during the second 5-year period). Kaplan-Meier estimation revealed a significant better survival after minor resections as compared to major and extended resections (Fig. 2). Median post-operative hospital stay over the entire study period was 9 days after minor resection, 11 days after major resection, and 13 days after extended resection, with no difference between the two study periods. The 30- and 90-day mortality figures, stratified for the extent of liver resection, are shown in detail in Table 1. The overall 30- and 90-day mortality rates were 1.8 and 3.1 %, respectively. There was no significant difference of postoperative mortality comparing the first with the second study period. In contrast, a significant difference was observed after hepatectomy performed in a non-university compared to university hospitals. In non-university hospitals, the 30- and 90-day mortality was 3.8 and 6.6 % and in university hospitals 1.6 and 2.8 %, respectively. The Kaplan-Meier estimation of survival after liver resection in non-university and university hospitals is shown in Fig. 3. In the majority of the cases (59 %), the indication for hepatic resection was liver metastasis (*n* = 2644). HCC was the diagnosis in 9 % (*n* = 393), GBC in 6 % (*n* = 254), ICC in 3 % (*n* = 129), and ECC in 2 % (*n* = 76). The remaining minor diagnosis groups were 2.5 % bowel cancer (without “metastasis”) (*n* = 110), 2 % other liver malignancies (*n* = 61), and 10 % other/unclear diagnosis (*n* = 452). Finally, about 8 % of the cases were found to have a benign diagnosis (*n* = 341). The 5-year overall survival rate was 50 % for the diagnosis “liver metastasis,” 40 % for HCC, 38 % for GBC, 30 % for ICC, and 20 % for ECC. The related Kaplan-Meier estimation is shown in Fig. 4.

**Table 1:** Postoperative 30- and 90-day mortality specified for the extent of hepatectomy

Extent of resection	n	Mortality %	
		30 days	90 days
Minor resections	2845 (63.8 %)	1.4	2.3
Major resections	1271 (28.5 %)	2.1	3.2
Extended resections	344 (7.7 %)	4.3	7.5

Minor resections (≤2 Couinaud segments), major resection (3–4 Couinaud segments), extended resection (>4 Couinaud segments)

**Fig. 5:**
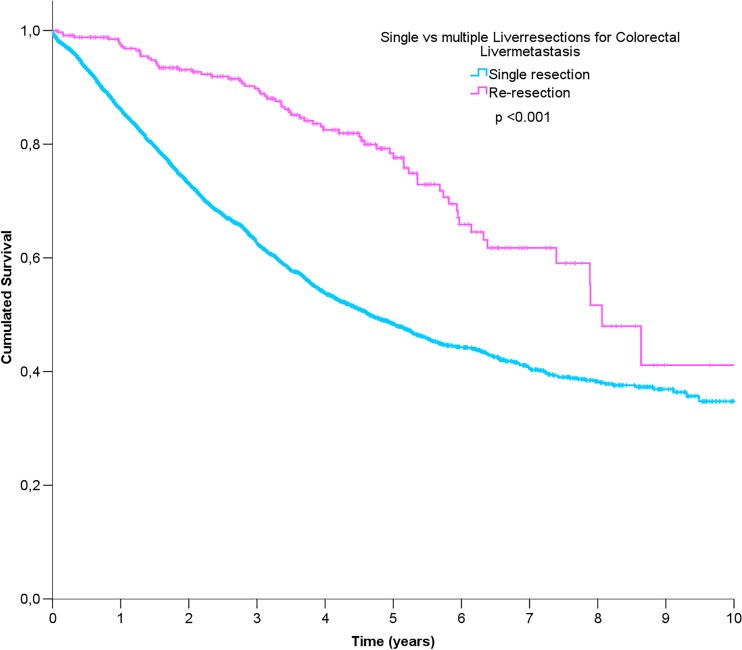
Kaplan-Meier survival estimates for patients with single and more than one liver resection for metastatic disease

Metastatic liver disease was the reason for 78 % of the re-resections. For this group of patients, the survival rate was significantly better after a second resection compared to those patients with a single-resection only (Fig. 5).

Univariable and multivariable risk factor analysis for the long-term outcome after liver resection is shown in detail in Table 2. High age, comorbidity, as defined by a modified Charlson score (described in the “Methods” section), male gender, and hepatectomy performed outside a university hospital were identified as independent risk factors for death. Low hospital volume was identified as a risk factor in the univariable analysis, too, but was excluded in the multivariable analysis due to co-linearity with the status “university/non-university hospital.” Accordingly, the diagnoses ICC, ECC, and GBC were identified as additional independent risk factors for death compared to patients with “liver metastasis” diagnosis. In addition, patients who underwent liver resection at non-university hospitals had a significantly decreased long-term survival rate compared with those who underwent surgery at university hospitals.

**Table 2:** Univariable and multivariable (Cox) regression analysis of risk factors for mortality (long-term survival)

	*n*	Univariable analysis		Multivariable analysis	
		HR (95 % CI)	*P*	HR (95 % CI)	*P*
Demography					
Age ≤ 54 years	1067	1.00		1.00	
Age 55–63 years	1143	1.39 (1.19–1.61)	<0.001	1.16 (0.99–1.35)	0.061
Age 64–71 years	1226	1.90 (1.65–2.20)	<0.001	1.55 (1.34–1.79)	<0.001
Age ≥ 72 years	1024	2.43 (2.11–2.80)	<0.001	1.90 (1.64–2.22)	<0.001
Female sex	2079	1.00		1.00	
Male sex	2381	1.20 (1.09–1.32)	<0.001	1.11 (1.01–1.22)	0.037
Comorbidity					
Charlson 0	3012	1.00		1.00	
Charlson 1–2	1388	1.44 (1.30–1.59)	<0.001	1.36 (1.22–1.50)	<0.001
Charlson 3–4	39	1.90 (1.26–2.87)	0.002	1.83 (1.20–2.78)	0.005
Charlson ≥5	21	3.23 (1.90–5.47)	<0.001	3.06 (1.80–5.21)	<0.001
Diagnosis					
CRCm	2644	1.00		1.00	
HCC	393	1.23 (1.06–1.44)	0.008	1.16 (0.99–1.36)	0.060
ICC	129	1.78 (1.41–2.25)	<0.001	1.76 (1.38–2.23)	<0.001
ECC	254	2.21 (1.70–2.93)	<0.001	2.15 (1.62–2.87)	<0.001
GBC	76	1.42 (1.19–1.70)	<0.001	1.53 (1.27–1.85)	<0.001
CRC	110	0.97 (0.73–1.30)	0.848	0.71 (0.52–0.95)	0.023
Other malignancy	61	0.83 (0.55–1.26)	0.384	0.83 (0.55–1.26)	0.391
Benign	341	0.15 (0.10–0.22)	<0.001	0.19 (0.13–0.28)	<0.001
Other	452	0.68 (0.57–0.81)	<0.001	0.74 (0.62–0.88)	0.001
Study period					
2002–2006	1619	1.00			
2007–2011	2841	1.05 (0.95–1.16)	0.393		
Extent of hepatectomy					
Minor	2845	1.00		1.00	
Major	1270	1.24 (1.12–1.38)	<0.001	1.18 (1.05–1.31)	0.004
Extended	345	1.64 (1.40–1.92)	<0.001	1.10 (1.28–1.78)	<0.001
Hospital volume					
High volume	3730	1.00			
Low volume	730	1.17 (1.05–1.32)	0.008		
Hospital structure					
University hospital	4096	1.00		1.00	
Non-university uospital	338	1.46 (1.26–1.69)	<0.001	1.57 (1.35–1.83)	<0.001
Re-resection					
No	4086	1.00		1.00	
Yes	374	0.40 (0.31–0.50)	<0.001	0.44 (0.34–0.56)	<0.001

*HR* hazard ratio, *95 % CI* 95 % confidence interval, *CRCm* colorectal cancer liver metastasis, *HCC* hepato-cellular carcinoma, *ICC* intra-hepatic cholangiocarcinoma, *ECC* extra-hepatic cholangiocarcinoma *GBC* gallbladder cancer, *CRC* diagnosis of colorectal cancer coded for liver resection but without diagnosis “metastasis,” other malignancy, other liver malignancies, other, other and unclear diagnosis

## Discussion

The present study demonstrates that hepatectomy is a safe procedure and that related mortality was probably overestimated in previous, non-population-based studies. The extent of resection and primary liver cancers are independent risk factors for post-operative mortality. Re-resection of liver metastasis improved long-term survival significantly. On the other hand, patients who underwent liver resections at non-university hospitals had a significantly worse outcome compared to those being resected at university hospitals.

The total number of hepatectomies in Sweden gradually increased during the study period (2002–2011). As expected, we observed huge differences in long-term outcomes depending on the underlying cancer diagnosis when adjusted for complementary risk factors like age and comorbidity, for example.

Previous publications addressing the outcome after liver surgery have mainly derived from single expert center experiences with a good chance of underestimating the risks and overestimating the results [15]. In Sweden, we have the opportunity to acquire medical information originating from the entire population with an almost complete follow-up using various national registries. An important methodological question emerges regarding the validity of these registers since the quality of studies like this is totally dependent on data quality and coverage. Previous validation studies have reported a 95 % accuracy concerning procedure codes in the Hospital Discharge Registry and a close to a 100 % registration in the Registry of Causes for Death and National Cancer Registry, respectively [21]. Therefore, it can be concluded that the registries used for this study are reliable.

We were able to demonstrate a low postoperative mortality after hepatic surgery and, as expected, a significant difference in immediate post-operative outcomes depending on the extent of hepatic resection. The 90-day mortality rate was about twice that of the 30-day mortality rate, which confirms previous observations regarding the limited value of reporting 30-day mortality figures only [22]. However, the mortality figures in Sweden differ from those reported by Farges et al. [17], who studied the 30- and 90-day mortality risks after liver resection in France between 2007 and 2009, and found them to be 3.4 and 5.8 %, respectively. This could partly be explained by a lower incidence of liver resections in Sweden compared to France, implying a more conservative selection of patients. Furthermore, HCC was not analyzed separately in the French study. It is reasonable to assume that hepatectomies due to HCC are more common in France than in Sweden, where only 9 % of all hepatectomies were performed for this indication. Moreover, in both countries, there might be a different case mix, too, that could influence the overall survival numbers. Despite an increase of major/extended liver resections of almost 100 % in the second study period, the postoperative mortality figures remained unchanged. The most likely explanation behind this favorable development is that the increase in the number of complex hepatectomies was exclusively seen at high volume university hospitals and as many as 99 % of major/extended hepatectomies were centralized to these units during the second study period. This development implies improved training of liver surgeons, something that has previously been reported as a major predictive factor for patient outcome after hepatectomy [5]. This conclusion is also supported by our finding that 30- and 90-day mortality is significantly higher for hepatectomies performed in low-volume, non-academic centers compared to university hospitals.

Regarding the long-term prognosis after surgery for primary and secondary liver cancers, we were able to define some independent risk factors for death. High age, severe comorbidity, and male gender were identified as patient-specific risk factors. Concerning the underlying cancer diagnosis, the highest risk of mortality was related to cholangiocarcinoma, gallbladder cancer, and hepatocellular cancer. In contrast, patients undergoing hepatectomy due to liver metastasis showed a significantly better 5-year survival, confirming earlier reports [8, 14, 23]. The significantly higher short-term mortality and worse long-term outcome after liver surgery in non-academic compared to academic units cannot be explained by, e.g., a higher frequency of synchronous procedures directed toward both the primary tumor and liver metastases but remained as an independent risk factor even in the multivariable analysis. Hence, it is tempting to speculate that surgeon/staff training and less radical surgery are reasons behind these results. Undoubtedly, these data further support the ongoing trend of centralization of corresponding surgical procedures to high volume centers.

In addition, we observed a significant survival benefit for patients after re-resection for liver metastasis. With this, we were able to confirm earlier reports deriving from single institutions in a population-based setting [24–27]. Given the possible impact of the selection of patients undergoing re-resection, our data provides evidence to justify an aggressive surgical approach in the individual patient and support the conclusion made by Antoniou and co-workers that re-resection should be encouraged whenever possible and feasible [9].

## Conclusion

In Sweden, liver surgery has expanded significantly during the last decade and is now practiced with a favorable postoperative risk profile. However, those with primary liver cancers still suffer a poor chance of long-term survival. Superior outcome of patients from University Hospitals support further centralization of liver surgery to high volume centers. In patients with liver metastases, re-resection should be performed if possible.

## Appendix

No. at risk.

**Table:**

Years	0	2	4	6	8	10
Minor	2845	1610	837	359	134	2
Major	1270	702	350	157	48	0
Extended	345	179	88	42	14	0

No. at risk.

**Table:**

Years	0	2	4	6	8	10
University	4096	2295	1160	490	164	2
Non-university	364	196	115	68	32	0

No. at risk.

**Table:**

Years	0	2	4	6	8	10
CRM	2644	1453	676	248	74	2
HCC	393	213	105	51	13	0
GBC	254	120	63	31	10	0
ICC	129	57	22	11	5	0
ECC	76	31	13	3	1	0

No. at risk.

**Table:**

Years	0	2	4	6	8	10
Single resection	2380	1261	562	209	64	1
Re-do resection	268	192	114	39	10	1
